# Second-Order Estimating Equations for Clustered Current Status Data from Family Studies Using Response-Dependent Sampling

**DOI:** 10.1007/s12561-017-9201-4

**Published:** 2017-07-24

**Authors:** Yujie Zhong, Richard J. Cook

**Affiliations:** 1 MRC Biostatistics Unit, School of Clinical Medicine, University of Cambridge, Cambridge Institute of Public Health, Forvie Site, Robinson Way, Cambridge, CB2 0SR UK; 2 Department of Statistics and Actuarial Science, University of Waterloo, Waterloo, ON N2L 3G1 Canada

**Keywords:** Current status data, Family study, Gaussian copula, Relative efficiency, Response-dependent sampling, Robustness, Second-order estimating equations

## Abstract

Studies about the genetic basis for disease are routinely conducted through family studies under response-dependent sampling in which affected individuals called probands are sampled from a disease registry, and their respective family members (non-probands) are recruited for study. The extent to which the dependence in some feature of the disease process (e.g., presence, age of onset, severity) varies according to the kinship of individuals reflects the evidence of a genetic cause for disease. When the probands are selected from a disease registry, it is common for them to provide quite detailed information regarding their disease history, but non-probands often simply provide their disease status at the time of contact. We develop conditional second-order estimating equations for studying the nature and extent of within-family dependence which recognizes the biased sampling scheme employed in family studies and the current status data provided by the non-probands. Simulation studies are carried out to evaluate the finite sample performance of different estimating functions and to quantify the empirical relative efficiency of the various methods. Sensitivity to model misspecification is also explored. An application to a motivating psoriatic arthritis family study is given for illustration.

**Electronic supplementary material:**

The online version of this article (doi:10.1007/s12561-017-9201-4) contains supplementary material, which is available to authorized users.

## Introduction

### Introduction

The heritable nature of disease can be inferred from the structure of the within-family dependence in disease manifestation [19]. For rare diseases, population-based cohort studies are inefficient and impractical, so response-dependent biased family designs are routinely employed to obtain enriched samples with higher representation of diseased individuals and more variation in genetic markers than would be seen in the unselected population. Much work has been carried out for the analysis of such data when the disease status is modeled as a binary trait. Conditional likelihood based on generalized linear mixed models can be used for dealing with the dependence of binary phenotypes within families [3, 4], or estimating equations can be formed by specifying marginal mean and dependence structures for the analysis of binary phenotypes from case–control family studies [32].

The age of onset for many chronic diseases is highly variable, however, and simply using the binary trait of the disease status does not account for the variable times individuals have been at risk for disease in family studies. MacLean et al. [21] and Shih and Chatterjee [27] pointed out that, if information on the age of onset and the effect of censoring are not addressed, the estimators of the covariate effects on the disease process may be less efficient and the degree of familial aggregation may be underestimated. Models which consider the disease onset time distribution and measure dependence in terms of these times offer a preferable framework for analysis.

When interest lies in examining genetic association or gene-environment interaction, case–control or case-only family study are commonly used. Li et al. [20], Hsu et al. [15], and Shih and Chatterjee [27] proposed likelihood methods based on disease onset time for case–control family study and Chatterjee et al. [6] proposed methods to estimate the relative risk, cumulative risk, and residual familial aggregation for case–control family data and modified method for case-only family data. In their methods, modeling and estimation of the residual familial aggregation is key to adjustment for ascertainment bias, but this is done using an exchangeable dependence structure in which the association is the same for different pairs of relatives. Gorfine et al. [14] use the frailty models to account for heterogeneity in familial risk, but pointed out that frailty-based methods may be affected by the uncertainty on the frailty parameter estimate.

In this article, we consider a simple family study, where an affected individual called a *proband* is selected from a registry of patients. Consenting family members (*non-probands*) of each proband are then recruited and examined to collect information on their disease status [5]. Probands are given a special designation because their disease status led to the selection of their family. Under such sampling scheme, one obtains a right-truncated onset time for the proband and current status (type I interval-censored) data for non-probands [28]. While work has been done on the analysis of multivariate current status data [8, 16], little has been done to our knowledge in the context of biased sampling schemes.

Insights into the genetic basis of the disease can be gained by comparing the strength of the association in disease status between pairs of family members with different kinships [7, 18]. More elaborate dependence modeling also plays a central role when studying the “parent-of-descent” hypothesis, where the primary goal is to estimate and compare the strength of father–child and mother–child associations in phenotype to elucidate the role of the sex chromosomes in disease transmission [30]. With this in mind, we consider copula models [22] as a basis for modeling the joint risk of disease among family members. The dependence parameters can be interpreted as reflecting “residual familial aggregation” that is not explained by covariates in the marginal models. Copula models have several advantages over frailty models. First, the marginal models still retain simple interpretation when using copula models, which is not the case under the frailty model. Second, copula models yield dependence measures which are functionally independent of the parameters in the marginal onset time distribution, so the marginal distribution can be specified in any desirable way. Third, the dependence measure is directly specified under the copula model which has clear meaning and it also provides a natural basis for regression of genetic effects, but the frailty models do not provide simple measures of within-family dependence and it is difficult to interpret the meaning of the dependence.

Analyses must address the biased sampling scheme employed in these studies. Likelihood contributions from each family which are proportional to joint probability functions for the phenotypes of non-probands *conditional* on the disease status of the proband will admit valid inference [29] under correct model specification, but enumeration of all possible sample outcomes can be computationally demanding with large families. We develop a class of conditional second-order estimating equations in the spirit of Prentice [25]. We use the term *conditional* to reflect the fact that moments in the second-order estimating equation are all conditional on the disease onset time of the proband. A supplementary estimating equation is incorporated to extract limited information about the marginal onset time distribution from the proband.

### The University of Toronto Psoriatic Arthritis Family Study

The incidence of psoriatic arthritis (PsA) is reported to be between 0.3 and 1.0% [9] and hereditary factors are thought to be important, as some studies have suggested that close blood relatives of individuals affected by psoriatic arthritis are at higher risk of developing the disease compared to the general population. Characterizing the within-family association nature and identifying important genetic risk factors are important to understand the disease etiology. Particular interest lies in assessing whether there is a higher rate of paternal, rather than maternal, transmission of the disease, which is also called “parent- of-origin” effect [2]. A family study of psoriatic arthritis is conducted in the Centre for Prognosis Studies in the Rheumatic Disease at the University of Toronto. Probands were selected from the members of the University of Toronto Psoriatic Arthritis Registry, and their family members were recruited into the family study with their consent. A total of 169 two-generation families ranging in size from 2 to 7 individuals were recruited; 54 families were comprised of only one non-proband and 115 have more than one non-proband. The disease onset times were only available for probands, but for other family members only the disease status is available when they are examined, yielding current status data. In total 538 individuals are in the family study and only 194 (169 probands and 25 non-probands) were diagnosed with PsA. Except for the demographic data, information of some HLA markers is also available for individuals in the PsA family study. We focus on identifying the significant HLA markers for the psoriatic arthritis and characterizing the within-family association structure, also testing whether there is “parent-of-origin” effect for the psoriatic arthritis.

The remainder of this paper is organized as follows. In Sect. 2 we define notation and formulate the conditional second-order estimating equation for family data under response-dependent sampling, which are a combination of right-truncated onset time from probands and current status data from non-probands. We consider an illustrative example in which the dependence structure is governed by a Gaussian copula and work with this model in subsequent calculations and simulations where we examine specific estimating equations involving different derivative matrices and working independence assumptions. In Sect. 3, we explore the asymptotic relative efficiencies and finite sample properties of estimators from several variants of the estimating equations introduced in Sect. 2; these results also permit sample size calculations for planning studies aiming to detect effects of genetic markers. The impact of misspecification of the dependence structure on properties of estimators and power of genetic tests is investigated in Sect. 4. An application to the motivating psoriatic arthritis family study is given in Sect. 5 in which we assess the genetic basis of the disease. Concluding remarks are given in Sect. 6.

## Conditional Estimating Equations Under Biased Sampling

### Notation, Sampling, and Observation Scheme for Family Studies

We consider the setting in which a registry of *M* individuals is created by selecting a random sample from a population, screening each individual for disease, and recruiting those found to have the condition of interest [10]. If $C_{i0}$ denotes the age of individual 0 in family *i* at the time of sampling and screening, and $T_{i0}$ denotes their age of disease onset, then this individual is recruited to the registry if $Y_{i0}=I(T_{i0} \le C_{i0})=1$; we assume that $T_{i0}$ is verifiable by a review of medical records for individuals recruited to the registry. When a family study is carried out, we assume that probands are selected from the disease registry by simple random sampling and without loss of generality we label the families of selected probands $i=1,\ldots , m$.

We let $T_{ij}$ and $X_{ij}$ denote, respectively, the event time and a $p \times 1$ covariate vector of individual *j* in family *i*, where $j= 1, \ldots , n_i$ are the labels for the non-probands. Then if $T_{i}=(T_{i0}, T_{i1}, \ldots , T_{in_{i}})'$ and $X_{i}=(X'_{i0}, \ldots , X'_{in_i})'$, we write the joint cumulative distribution function (j.c.d.f) for family *i* as $F_i(t)= P(T_{i0} \le t_0, \ldots , T_{in_i} \le t_{n_i}|X_i)$. We assume $T_{ij} \perp X^{(-j)}_{i}|X_{ij}$, where $X_{i}^{(-j)} = \{X_{ij'}; j'\ne j, 0 \le j' \le n_i\}$, and write $F_{ij}(t;\theta ) = P(T_{ij} \le t|X_{ij}; \theta )$. The marginal hazard function for the disease onset time of individual *j*, $j=0, 1, \ldots , n_i$, in family *i* is

$$\begin{aligned} \lambda _{ij}(t|X_{ij}; \theta ) = \lim _{\Delta t \downarrow 0} \frac{P(t \le T_{ij} < t +\Delta t|t \le T_{ij}, X_{ij};\theta )}{\Delta t}, \end{aligned}$$

where we write $\lambda _{ij}(t|X_{ij};\theta ) = \lambda _0 (t; \alpha ) \exp (X'_{ij}\beta )$ under a proportional hazards formulation. This gives $F_{ij}(t_{ij};\theta ) = 1- \exp ( - \Lambda _0 (t_{ij}; \alpha ) \exp (X'_{ij}\beta ) )$, where $\Lambda _0 (t_{ij}; \alpha ) = \int _0^{t_{ij}} \lambda _0 (s; \alpha ) \mathrm{d}s$, $\alpha$ is a $q \times 1$ vector, $\beta$ is a $p \times 1$ vector of regression coefficients, and $\theta =(\alpha ',\beta ')'$. We let $\gamma$ parameterize the within-family dependence and $\psi = (\theta ' , \gamma ' )'$.

Classification of non-probands with respect to their disease status is made at the time of recruitment and clinical examination, yielding current status data. Let $C_{ij}$ denote the age of non-proband *j* in family *i* at the time of assessment and let $Y_{ij} = I(T_{ij} \le C_{ij})$; we let $\bar{C}_i = (C_{i1},\ldots , C_{in_i})'$, $\bar{Y}_i = (Y_{i1}, \ldots , Y_{in_i})'$ and $\bar{X}_i = (X'_{i1}, \ldots , X'_{in_i})'$. If $Y_i = (Y_{i0}, \bar{Y}'_i)'$, $C_i = (C_{i0}, \bar{C}'_i)'$, and $X_i = (X'_{i0}, \bar{X}'_i)'$, the family data therefore consist of $\{T_{i0}, Y_i, C_i, X_i \}$ subject to $Y_{i0}=1$.

### Second-Order Estimating Functions

The association parameter $\gamma$ is of central importance here so we next formulate conditional second-order generalized estimating equations in the spirit of Prentice [25] and Zhao and Prentice [33].

Let $\bar{Z}_{i} = (Y_{i1}Y_{i2}, Y_{i1}Y_{i3}, \ldots , Y_{i1}Y_{i n_{i}}, Y_{i2}Y_{i3}, \ldots , Y_{i,n_{i}-1}Y_{in_{i}})'$ be an $r_i\times 1$ vector of pairwise products of the elements in $\bar{Y}_{i}$, where $r_i=n_{i}(n_{i}-1)/2$; we let $Z_{ijk}$ denote the element of $\bar{Z}_i$ corresponding to the pair (*j*, *k*) in family *i*. To account for response-biased sampling, we define conditional moments and let $\mu _{i} = E[\bar{Y}_{i}|T_{i0};\psi ]$ and $\eta _i = E[\bar{Z}_i | T_{i0};\psi ]$ be the contributions from the non-probands and let $\mu _{i0} = E[T_{i0}|Y_{i0} = 1;\theta ]$ for the proband where we suppress the dependence on $X_i$ and $C_i$. The conditional second-order estimating equations (CGEE2) denoted by $U(\psi ) = \sum _{i=1}^{m} U_i (\psi ) =0$ have the form:

1 $$\begin{aligned} U_{i}(\psi ) = G'_i\, W^{-1}_i R_i + D'_i \, V^{-1}_i (T_{i0} - \mu _{i0}) \end{aligned}$$

with

$$\begin{aligned} G_i= & {} \left( \begin{array}{cc} G_{i11} &{} G_{i12} \\ G_{i21} &{} G_{i22}\end{array}\right) , ~~ W_i = \left( \begin{array}{cc} W_{i11} &{} W_{i12} \\ W'_{i12} &{} W_{i22}\end{array}\right) , ~~\mathrm{and}~~ R_i = \left( \begin{array}{c} \bar{Y}_i - \mu _i \\ \bar{Z}_i - \eta _i \end{array}\right) , \end{aligned}$$

where $G_{i11} = \partial \mu _i /\partial \theta '$, $G_{i12} = \partial \mu _i /\partial \gamma '$, $G_{i21} = \partial \eta _i /\partial \theta '$, and $G_{i22} = \partial \eta _i /\partial \gamma '$, $W_{i11} = \mathrm{Cov}(\bar{Y}_i, \bar{Y}'_i|T_{i0})$, $W_{i22} = \mathrm{Cov}(\bar{Z}_i, \bar{Z}'_i|T_{i0})$, and $W_{i12} = \mathrm{Cov}(\bar{Y}_i, \bar{Z}'_i|T_{i0})$ ; note that unlike standard GEE2, $G_{i12}\ne \varvec{0}$ since $\mu _i = E[\bar{Y}_i|T_{i0};\psi ]$ is functionally dependent on $\gamma$. The covariance matrices can be parameterized by the marginal and association parameters where the latter may be specified in terms of Kendall’s $\tau$; an example is given in Sect. 2.3. Consistent estimation of $\psi$ is possible based on the first term in (1), but the second term $D'_i V^{-1}_i (T_{i0} - \mu _{i0})$, where $D_i = \partial \mu _{i0}/\partial \psi '$ and $V_i = \mathrm{Var}(T_{i0}|Y_{i0}=1)$, improves efficiency by exploiting the data on the onset time from the proband.

Subject to correct specification of the conditional moments, (1) is an unbiased estimating function, so the estimator $\widehat{\psi }$ solving $U(\psi ) = 0$ is consistent with an asymptotic normal distribution

2 $$\begin{aligned} \sqrt{m} (\widehat{\psi } - \psi ) \xrightarrow {~d~} N\left( 0, \mathcal{A}^{-1}(\psi ) \mathcal{B}(\psi ) \left[ \mathcal{A}^{-1}(\psi )\right] ' \right) , \end{aligned}$$

where

$$\begin{aligned} \mathcal{A}(\psi )= & {} E[-\partial U_i (\psi ) /\partial \psi \,']~~~\mathrm{and}~~~\mathcal{B}(\psi ) = E[U_i (\psi ) U'_i (\psi )]. \end{aligned}$$

Natural empirical estimates of these matrices are

3 $$\begin{aligned} A(\widehat{\psi }) = \frac{1}{m}\sum _{i=1}^{m} \left\{ \widehat{G}'_i\, \widehat{W}_i^{-1} \widehat{G}_i + \widehat{D}'_i \, \widehat{V}_i^{-1} \widehat{D}_i\right\} \end{aligned}$$

and

$$\begin{aligned} B(\widehat{\psi })= & {} \frac{1}{m}\sum _{i=1}^{m}\bigg \{\widehat{G}'_i\, \widehat{W}^{-1}_i \widehat{R}_i \widehat{R}'_i \widehat{W}_{i}^{-1} \widehat{G}_i + \widehat{D}'_i\, \widehat{V}^{-1}_{i}(T_{i0}-\widehat{\mu }_{i0})^2 \widehat{V}_{i}^{-1}\widehat{D}_i\bigg \},\nonumber \end{aligned}$$

which yield $$\widehat{\mathrm{asvar}}(\sqrt{m}(\widehat{\psi }-\psi )) = A^{-1}(\widehat{\psi }) B(\widehat{\psi }) [A^{-1}(\widehat{\psi })]'$$.

Simplified forms of $G_i$ can be obtained by setting $G_{i21}=\partial \eta _i/\partial \theta '= \varvec{0}$ (denoted by $G^{\mathrm{I}}$) or by letting both $G_{i12} = \partial \mu _i/ \partial \gamma ' = \varvec{0}$ and $G_{i21} = \partial \eta _i /\partial \theta ' = \varvec{0}$ (denoted by $G^\mathrm{II}$). It is also common to simplify $W_i$ and adopt a form in which $W_{i12} = W'_{i21} = \mathbf{0}$ and $W_{i22} = \mathrm{diag}\{\eta _i (1-\eta _i)\}$ while retaining the full structure of $W_{i11}$; we refer to this as a *working partial independence* (WPI) matrix. Combining these simplifications, we consider four different estimating functions based on (1)

A. Full $G_i$ and Full covariance matrix $W_i$ denoted G–W,
B. Full $G_i$ and WPI $W_i$ denoted G-WPI,
C. G$^{\mathrm{I}}$ and WPI $W_i$ denoted G$^{\mathrm{I}}$-WPI, and
D. G$^{\mathrm{II}}$ and WPI $W_i$ denoted $G^{\mathrm{II}}$-WPI.

### An Illustrative Dependence Structure Based on a Gaussian Copula

The specific form of the moments for $T_i | T_{i0}, X_i$ can be motivated by a copula model. Consider an $(n_i+1) \times 1$ vector of uniform [0, 1] variables $K_i = (K_{i0}, K_{i1}, \ldots , K_{in_i})'$, in which $K_{ij} = F_{ij}(t_{ij};\theta )$, $j=0,\ldots , n_i$. The j.c.d.f. for $K_i$, denoted by $H_{n_i+1}(k;\gamma ) = P(K_{i0} \le k_{i0}, K_{i1} \le k_{i1}, \ldots , K_{i n_i} \le k_{i n_i}; \gamma )$, is a copula function in $n_i+1$ dimensions indexed by an $r\times 1$ parameter $\gamma$ which characterizes the dependence [17, 22]. The Gaussian copula is a member of elliptical family of the form:

$$\begin{aligned} H_{n_i+1}(k_{i0}, \ldots , k_{in_i}; \gamma )=\Phi _{n_i+1}\big (\Phi ^{-1}(k_{i0}), \ldots , \Phi ^{-1}(k_{in_i}); \gamma \big ), \end{aligned}$$

where $\Phi ^{-1}(\cdot )$ is the inverse cumulative distribution function of a standard normal random variable (r.v.) and $\Phi _{n_i + 1}(\cdot \, ; \gamma )$ is the j.c.d.f. of an $(n_i + 1) \times 1$ multivariate normal r.v. with mean zero and $(n_i + 1) \times (n_i + 1)$ correlation matrix $\Sigma _i$; $\Sigma _i$ is indexed by $\gamma$ and we denote the off-diagonal entries by $\sigma _{ijk}$, $j \ne k=0,\ldots , n_i$. Specification of the Gaussian copula for $K_i$ induces a joint distribution for $T_i | X_i$ given by

4 $$\begin{aligned} P(T_{i0} \le t_{i0}, \ldots , T_{in_{i}} \le t_{i n_i}|X_i; \psi )\!\! =\!\! \int _{-\infty }^{q_{i0}}\cdots \int _{-\infty }^{q_{in_{i}}} \frac{\exp (-s'_i\Sigma _{i}^{-1}s_{i} / 2)}{\sqrt{(2\pi )^{n_{i} \!+\! 1}|\Sigma _{i}|}}\mathrm{d}s_{i0}\cdots \mathrm{d}s_{in_{i}}, \end{aligned}$$

where $S_{i}\sim \mathrm{MVN}_{n_i + 1}(0, \Sigma _i )$, $s_i$ is a realization, and $q_{ij}=\Phi ^{-1}(F_{ij}(t_{ij}; \theta ))$, $j=0,\ldots , n_{i}$. Copula functions such as this are attractive for dependence modeling since pairwise associations are parameterized to be functionally independent of the marginal parameters and different pairwise associations are permitted. The Kendall’s $\tau$ characterizing the association between $T_{ij}$ and $T_{ik}$ given $X_{i}$, for example, is given by $\tau _{ijk}= 2*arcsin(\sigma _{ijk})/\pi$, $0\le j < k \le n_i$. Regression modeling of the within-family dependence can be achieved by specifying a second-order model of the form $g(\tau _{ijk}) = v_{ijk}'\gamma$, where $g(\cdot )$ is a 1–1 differentiable link function mapping Kendall’s $\tau$ onto the real line, $v_{ijk}$ is an $r\times 1$ covariate vector characterizing individuals *j* and *k* in family *i* and their relationship, and $\gamma$ is the corresponding $r\times 1$ vector of coefficients. This second-order regression model can be helpful when investigating the effect of risk factors on the pairwise association as $v_{ijk}$ could represent family-level or individual-level features, or information on the kinship of individuals *j* and *k* in family *i*; inference on their effects can be easily carried out based on $\gamma$. For example, in the PsA family study with two generations, when the “parent-of-origin” hypothesis is of interest, we can formulate the second-order model as

$$\begin{aligned} g(\tau _{ijk})= & {} \gamma _0 + \gamma _1 \, \text{ I }((j, k)~\text{ pair } \text{ are } \text{ siblings }) + \gamma _2 \, \text{ I }((j, k)~\text{ pair } \text{ is } \text{ father--child }) \\&+ ~ \gamma _3 \, \text{ I }((j, k)~\text{ pair } \text{ is } \text{ mother--child }), \end{aligned}$$

then comparing $\gamma _2$ and $\gamma _3$ (or testing $H_0 : \gamma _2 = \gamma _3$) can inform us whether there is “parent-of-origin” effect in the onset time of PsA. More elaborate models which incorporate genetic covariates into the dependence model can also be specified.

Returning to the estimating function in (1), based on the Gaussian copula we have $\mu _{ij} = E[Y_{ij}|T_{i0}] = P(T_{ij} \le C_{ij}|T_{i0}) = \Phi ((q_{ij} - \sigma _{i0j}q_{i0})/(1-\sigma ^2_{i0j})^{1/2})$ and $\eta _{ijk}= E[Y_{ij}Y_{ik}|T_{i0}] = \Phi _2 \left( (q_{ij} - \sigma _{i0j}q_{i0}), (q_{ik} - \sigma _{i0k}q_{i0}); \Sigma _{jk|0}\right)$, where $q_{ij} = \Phi ^{-1}(F_{ij}(C_{ij}))$, $j=1,\ldots , n_i$, and $q_{i0} = \Phi ^{-1}(F_{i0}(t_{i0}))$. The function $\Phi _2 (\cdot , \cdot \,; \Sigma _{jk|0})$ is the j.c.d.f of a bivariate normal r.v. with mean zero and covariance matrix $\Sigma _{jk|0}$, where

$$\begin{aligned} \Sigma _{jk|0} = \left( \begin{array}{cc} 1-\sigma _{i0j}^2 &{} \sigma _{ijk}-\sigma _{i0j}\sigma _{i0k} \\ \sigma _{ijk}-\sigma _{i0j}\sigma _{i0k} &{} 1- \sigma _{i0k}^2 \end{array}\right) . \end{aligned}$$

The entries of $W_i$ can also be derived based on the Gaussian copula where, for example, $\mathrm{cov}(Y_{il}, Z_{ijk}|T_{i0}) = E[Y_{il}Y_{ij}Y_{ik}|T_{i0}] - \mu _{il}\eta _{ijk}$ for $k\ne l\ne j$ with

$$\begin{aligned} E[Y_{il}Y_{ij}Y_{ik}|T_{i0}]= & {} \phi ^{-1}(q_{i0})\cdot \int _{-\infty }^{q_{il}}\int _{-\infty }^{q_{ij}}\int _{-\infty }^{q_{ik}} \phi _4 \big (q_{i0}, s_{il}, s_{ij}, s_{ik}\, ;\; \Sigma _i (0, l, j, k)\big )\\&\quad \mathrm{d}s_{ik}\mathrm{d}s_{ij}\mathrm{d}s_{il}. \end{aligned}$$

Note that this is a j.c.d.f for a multivariate normal r.v. with mean $\mu ^\dagger$ and covariance matrix $\Gamma ^\dagger$ denoted by $\Phi _3 (q_{il}, q_{ij}, q_{ik}\, ; \; \mu ^\dagger , \Gamma ^\dagger )$, where $\mu ^\dagger \! \!=\! (\sigma _{i0l}q_{i0}, \sigma _{i0j}q_{i0}, \sigma _{i0k}q_{i0})'$ and

$$\begin{aligned} \Gamma ^\dagger = \left( \begin{array}{ccc} 1-\sigma ^2_{i0l} &{} \sigma _{ilj}-\sigma _{i0l}\sigma _{i0j} &{} \sigma _{ilk} - \sigma _{i0l}\sigma _{i0k}\\ \sigma _{ilj} - \sigma _{i0l}\sigma _{i0j} &{} 1-\sigma ^2_{i0j} &{} \sigma _{ijk} - \sigma _{i0j}\sigma _{i0k} \\ \sigma _{ilk} - \sigma _{i0l}\sigma _{i0k} &{} \sigma _{ijk}-\sigma _{i0j}\sigma _{i0k} &{} 1-\sigma _{i0k}^2 \end{array}\right) . \end{aligned}$$

These conditional moments are easily derived under a Gaussian copula.

## Relative Efficiency Under Particular Estimating Equations

### A Study of Asymptotic Relative Efficiency

Here we examine the asymptotic relative efficiency of four different conditional estimating equations as a function of the strength of the within-family association through the functions:

$$\begin{aligned} \mathrm{ARE}_{\mathrm{B}} (\widehat{\psi }) = \frac{\mathrm{asvar_{\mathrm{A}}} (\widehat{\psi })}{\mathrm{asvar_{\mathrm{B}}} (\widehat{\psi })} \; ,\mathrm{ARE}_{\mathrm{C}} (\widehat{\psi }) \!=\! \frac{\mathrm{asvar_{\mathrm{A}}} (\widehat{\psi })}{\mathrm{asvar_{\mathrm{C}}} (\widehat{\psi })} , \mathrm{and}\mathrm{ARE}_{\mathrm{D}} (\widehat{\psi }) = \frac{\mathrm{asvar_{\mathrm{A}}} (\widehat{\psi })}{\mathrm{asvar_{\mathrm{D}}} (\widehat{\psi })} , \end{aligned}$$

where $\mathrm{asvar}( )$ denotes an asymptotic variance and its subscript indexes the adopted conditional estimating equations proposed in Sect. 2.2 . All three simplified conditional estimating equations are compared with the conditional estimating equations with full $G_i$ and full covariance matrix $W_i$.

Consider two-generation families composed of two parents and two children, $n_i = 3$. The proband is randomly selected from the four family members and is indexed by $j=0$. A Weibull distribution is adopted for the onset time for all family members; $\mathcal{F}(t_{ij}|X_{ij}; \theta ) = \exp \big (-(\lambda t_{ij})^\kappa \exp (X_{ij}\beta )\big )$, where $X_{ij}$ is a binary variable with $P(X_{ij} = 1) = 0.5$, $j=0, 1, 2, 3$, and we assume that $X_{ij} \perp X_{ik}$, $j \ne k$; $\theta =(\lambda , \kappa , \beta )'$. Let $\kappa = 1.2$, $\beta = \log 1.2$, and choose $\lambda$ to give a median age of 45 years for disease onset for group with $X_{ij}=0$. The clinic entry time for the proband $C_{i0}$ is normally distributed with mean 50 and variance 20, and families are recruited into the study only if their probands satisfy the selection condition $T_{i0} \le C_{i0}$. For non-proband *j* in the selected family *i*, let $C_{ij}$ be the random age of contact, following $N(\mu =60, \sigma ^2 = 10)$ for individuals in the first generation and $N(\mu =30, \sigma ^2 = 10)$ for the individuals in the second generation, $j=1, 2, 3$; the age at contact for individuals in both generation are truncated at 90 years. We consider a Gaussian copula to induce an exchangeable within-family association for simplicity here, and let Kendall’s $\tau$ vary from 0 to 0.5 to reflect independence to strong within-family association. The second-order model with a Fisher transformation link function is simply $\log \big ((1+\tau _{ijk})/(1-\tau _{ijk})\big ) = \gamma _0$, $0\le j < k \le 3$. The asymptotic variances of estimators based on conditional estimating equations in (2) are approximated by Monte Carlo simulation based on 20,000 samples.

**Fig. 1:**
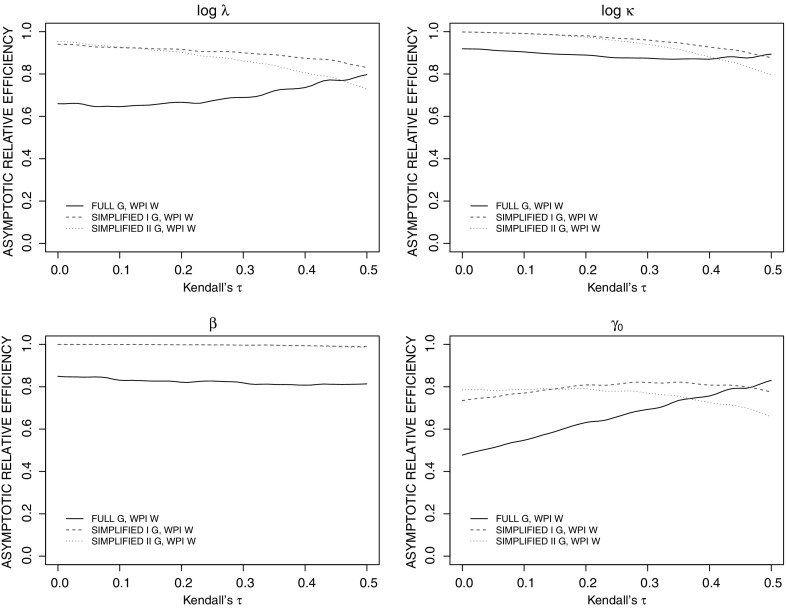
Asymptotic relative efficiencies of estimators under conditional estimating equations with full $G_i$ and WPI $W_i$ ($\mathrm{ARE}_\mathrm{B}$), simplified G$^\mathrm{I}$ and WPI $W_i$ ($\mathrm{ARE}_\mathrm{C}$), simplified G$^\mathrm{II}$ and WPI $W_i$ ($\mathrm{ARE}_\mathrm{D}$) compared with that under conditional estimating equation with full $G_i$ and full covariance matrix $W_i$; within-family dependence of disease onset times is induced by a Gaussian copula with exchangeable structure with Kendall’s $\tau$ varying from 0 to 0.5; $(\log \lambda , \log \kappa , \beta ) = (-4.11, \log 1.2, \log 1.2)$, $n_i = 3$, $m=20{,}000$

Figure 1 shows the trends of asymptotic relative efficiencies of estimators under different conditional estimating equations as a function of the within-family association. It is apparent that the conditional estimating equations with full $G_i$ and full $W_i$ (G–W) lead to the most efficient estimators, and the efficiency gain is most appreciable for the association parameter. With the WPI matrix $W_i$, adopting $G^\mathrm{I}$ yields more efficient estimators than that using $G^\mathrm{II}$, especially when the within-family association is strong. This makes sense as the former utilizes additional information about $\gamma$ from the conditional mean $\mu _i$. The conditional estimating equations with the full $G_i$ and WPI matrix $W_i$ (G-WPI) perform worse than other approaches when the association is less than 0.45; see Fig. 1. This indicates that with a working covariance matrix, using the full derivative matrix increases the complexity, but does not improve efficiency; on the contrary, it leads to less efficient estimators. This is similar to the findings reported by Balemi and Lee [1] where they compare the performance of GEE1 and GEE2 estimators for clustered binary data.

### Finite Sample Study of the Conditional Estimating Equations

Here we conduct a simulation study to assess the validity and finite sample performance of these four conditional estimating equations for family data from response-dependent sampling. The parameter settings are the same as in Sect. 3.1 and we let Kendall’s $\tau = 0.0, 0.2$, and 0.4 for an exchangeable Gaussian copula. We also consider a more general case where the within-family association is induced by a Gaussian copula with structured correlation matrix. For the two-generation families composed of two parents and two children, let Kendall’s $\tau _\mathrm{pp} = 0.1$ for parents, Kendall’s $\tau _\mathrm{pc} = 0.2$ for parent–child, and Kendall’s $\tau _\mathrm{ss}=0.4$ for siblings to reflect the existence of both environmental and genetic effects on the age of onset. One thousand datasets of $m=200$ and 1000 ascertained families are generated, the four proposed conditional estimating equations are used for analysis, and the empirical properties of estimates of $\beta$ and $\gamma _0$ are summarized in Table 1 for the exchangeable Gaussian copula and $\beta$ and $\gamma =(\gamma _0,\gamma _1,\gamma _2)'$ for the structured Gaussian copula in Table 2. The performance of the estimators for the parameters of the baseline hazard was excellent under the (correct) Weibull specification in all settings and so we do not tabulate these results.

**Table 1:** Empirical properties of estimates under conditional estimating equations for family data from response-dependent sampling, where within-family association is induced by Gaussian copula with exchangeable structure; parametric margins and piecewise constant baseline hazards (3 pieces) are considered$^\mathrm{a}$; $n_i = 3$, $nsim=1000$

		Weibull margin								Piecewise constant margin$^\mathrm{a}$								
		$\beta$				$\gamma _0$				$\beta$				$\gamma _0$				
$\tau$	EE$^\mathrm{b}$	EBIAS	ESE	ASE	ECP%	EBIAS	ESE	ASE	ECP%	EBIAS	ESE	ASE	ECP%	EBIAS	ESE	ASE	ECP%	NC %
$$m=200$$																		
0.0	G–W	0.002	0.113	0.115	95.5	-0.002	0.067	0.065	94.1	0.002	0.113	0.115	95.7	-0.000	0.070	0.067	93.4	0.2
	G-WPI	0.001	0.124	0.125	95.7	0.000	0.100	0.096	93.9	0.002	0.125	0.125	95.7	0.004	0.111	0.102	94.0	1.3
	G$^{\mathtt{I}}$-WPI	0.001	0.113	0.115	95.7	-0.001	0.080	0.077	93.2	0.002	0.114	0.115	95.6	0.000	0.084	0.080	93.7	0.0
	G$^{\mathtt{II}}$-WPI	0.001	0.113	0.115	95.7	-0.001	0.078	0.074	93.2	0.001	0.113	0.115	95.7	0.001	0.081	0.076	93.2	0.2
0.2	G–W	-0.006	0.109	0.109	94.5	0.002	0.106	0.105	94.6	-0.006	0.109	0.108	94.3	0.021	0.115	0.112	95.2	0.1
	G-WPI	-0.010	0.120	0.119	95.1	0.009	0.136	0.133	95.0	-0.011	0.120	0.118	95.1	0.050	0.163	0.151	93.8	2.2
	G$^{\mathtt{I}}$-WPI	-0.006	0.109	0.109	94.2	0.005	0.118	0.117	94.8	-0.007	0.109	0.108	94.2	0.032	0.132	0.126	95.0	0.0
	G$^{\mathtt{II}}$-WPI	-0.007	0.109	0.109	94.3	0.006	0.119	0.119	95.4	-0.007	0.108	0.108	94.1	0.031	0.133	0.128	95.8	0.1
0.4	G-W	0.001	0.092	0.094	96.0	0.008	0.152	0.154	95.6	-0.003	0.090	0.092	96.3	0.065	0.174	0.164	94.3	0.1
	G-WPI	0.001	0.103	0.104	96.4	0.015	0.173	0.175	95.7	-0.005	0.101	0.101	95.8	0.100	0.208	0.197	93.3	1.6
	G$^{\mathtt{I}}$-WPI	0.001	0.093	0.094	96.0	0.013	0.170	0.173	96.6	-0.004	0.091	0.092	95.6	0.083	0.195	0.189	96.6	0.0
	G$^{\mathtt{II}}$-WPI	0.000	0.093	0.094	96.1	0.016	0.183	0.186	96.0	-0.004	0.091	0.092	95.7	0.091	0.217	0.211	98.4	2.0
$$m=1000$$																		
0.0	G–W	-0.001	0.051	0.052	95.6	0.001	0.030	0.029	94.7	-0.002	0.049	0.052	95.9	0.001	0.030	0.030	95.3	0.0
	G-WPI	-0.001	0.055	0.056	95.4	-0.000	0.042	0.043	95.1	-0.002	0.054	0.056	95.6	0.001	0.046	0.046	94.9	0.0
	G$^{\mathtt{I}}$-WPI	-0.001	0.051	0.052	95.8	-0.000	0.035	0.035	94.0	-0.002	0.049	0.052	96.0	0.001	0.036	0.036	94.9	0.0
	G$^{\mathtt{II}}$-WPI	-0.001	0.051	0.052	95.8	0.000	0.034	0.034	93.9	-0.002	0.049	0.052	96.1	0.002	0.035	0.034	95.1	0.0
0.2	G–W	-0.004	0.050	0.049	93.2	0.002	0.048	0.047	95.2	-0.005	0.050	0.048	93.4	0.021	0.051	0.050	93.5	0.0
	G-WPI	-0.004	0.054	0.054	93.6	0.004	0.059	0.059	95.6	-0.005	0.054	0.053	93.5	0.039	0.068	0.067	92.7	0.0
	G$^{\mathtt{I}}$-WPI	-0.004	0.050	0.049	93.4	0.003	0.053	0.052	95.0	-0.005	0.050	0.048	93.4	0.031	0.057	0.056	93.2	0.0
	G$^{\mathtt{II}}$-WPI	-0.004	0.050	0.049	93.4	0.004	0.054	0.053	94.7	-0.005	0.050	0.048	93.4	0.030	0.058	0.057	92.8	0.0
0.4	G–W	0.000	0.042	0.042	95.0	0.003	0.068	0.068	95.0	-0.004	0.041	0.041	94.7	0.058	0.076	0.073	88.9	0.0
	G-WPI	-0.000	0.045	0.047	95.5	0.006	0.078	0.077	95.1	-0.007	0.044	0.045	95.3	0.088	0.092	0.088	83.7	0.0
	G$^{\mathtt{I}}$-WPI	0.000	0.042	0.042	95.0	0.005	0.075	0.075	95.9	-0.005	0.041	0.041	94.7	0.076	0.085	0.083	86.7	0.0
	G$^{\mathtt{II}}$-WPI	-0.000	0.042	0.042	94.9	0.005	0.080	0.080	96.0	-0.005	0.041	0.041	94.8	0.079	0.094	0.090	88.6	0.0

$^\mathrm{a}$ Empirical properties are summarized based on replicates leading to convergence of the piecewise constant model. The percentages of replicates failing to converge (out of $nsim=1000$) are indicated in the last column (all replicates converged for the approach based on the correct Weibull marginal model)

$^\mathrm{b}$ G$^\mathtt{I}$ corresponds to $G_i$ with $G_{i21}=\partial \eta _i /\partial \theta ' = 0$; G$^\mathtt{II}$ corresponds to $G_i$ with $G_{i12} = 0$ also; WPI represents the working partial independence assumption with $W_{i22} = \mathrm{diag}\{\eta _i (1-\eta _i)\}$, $W_{i12} = W'_{i21} = 0$

The results under the exchangeable Gaussian copula in Table 1 show that when the Weibull model is specified for the onset time distribution, the empirical biases are negligible for all conditional estimating equations; there is very slight finite sample bias for the association parameter when $m=200$ and the within-family association is strong (Kendall’s $\tau =0.4$). The empirical standard errors (ESE) agree with the average standard errors (ASE) based on the robust variance form, and the empirical coverage probabilities (ECP) of nominal 95% confidence intervals are in general within the acceptable range. Consistent with the theoretical results of Sect. 3.1, the greatest efficiency came from the conditional estimating equations with the full derivative matrix and full covariance matrix (G–W), followed by those with G$^\mathrm{I}$ and WPI matrix $W_i$ (G$^\mathrm{I}$-WPI). The empirical performance of the conditional estimating equations with the full $G_i$ and WPI matrix $W_i$ is worse than the others, again in alignment with the conclusion based on Fig. 1.

When considering a more flexible marginal model with a piecewise constant (3 pieces) baseline hazard function, we set the cut-points at $t=20$ and 40. For the large sample size $m=1000$, performance was excellent for inference regarding $\beta$ and very good for the dependence parameter $\gamma _0$ when the association was small; properties of the estimator of $\gamma _0$ became worse with stronger within-family dependence, possibly as a result of the crude approximation of the piecewise constant hazard. While one might expect superior performance if more pieces were accommodated, convergence problems arose even with just three pieces under the smaller sample sizes for some replicates (typically less than 2.5%); the percentages of replicates failing to converge are reported in the last column and where necessary the properties of estimators from converged replicates are given. The G$^\mathrm{I}$-WPI estimating equation always resulted in convergence. The convergence issues likely arose due to the right-truncated nature of the proband onset time and the severe censoring from a current status observation scheme of non-probands; these combine to yield little information to estimate the hazard function in small samples.

**Table 2:** Empirical properties of estimates under conditional estimating equations for family data from response-dependent sampling, where within-family association is induced by Gaussian copula with structure, $\tau _{pp} = 0.1, \tau _{ss}=0.4$ and $\tau _{pc} = 0.2$; parametric margins and piecewise constant baseline hazards (3 pieces) are considered$^\mathrm{a}$; $n_i = 3$, $nsim=1000$

		$\beta$				$\gamma _0$				$\gamma _1$				$\gamma _2$				
Margins	EE$^\mathrm{b}$	BIAS	ESE	ASE	ECP%	BIAS	ESE	ASE	ECP%	BIAS	ESE	ASE	ECP%	BIAS	ESE	ASE	ECP%	NC %
$$m=200$$																		
Weibull	G-W	0.001	0.103	0.104	96.3	-0.002	0.172	0.169	94.4	0.015	0.194	0.196	95.2	0.004	0.143	0.138	94.7	0.0
	G-WPI	0.001	0.115	0.114	95.3	0.006	0.239	0.233	94.2	0.015	0.267	0.262	94.3	0.001	0.196	0.191	94.2	0.0
	G$^{\mathtt{I}}$-WPI	0.000	0.103	0.104	96.3	-0.001	0.216	0.210	93.3	0.018	0.262	0.256	94.8	0.004	0.193	0.187	94.6	0.0
PWC-3$^\mathrm{a}$	G-W	-0.000	0.102	0.103	96.2	0.011	0.179	0.175	94.4	0.031	0.207	0.208	95.0	0.011	0.147	0.142	94.3	0.2
	G-WPI	-0.001	0.114	0.112	95.1	0.032	0.255	0.246	93.7	0.058	0.317	0.303	94.0	0.016	0.211	0.205	94.3	3.9
	G$^{\mathtt{I}}$-WPI	-0.001	0.102	0.103	96.3	0.013	0.227	0.219	93.3	0.051	0.298	0.286	94.8	0.016	0.205	0.197	94.7	0.4
$$m=1000$$																		
Weibull	G-W	-0.002	0.045	0.047	95.9	-0.000	0.073	0.075	97.2	0.006	0.084	0.088	96.2	0.001	0.060	0.062	95.2	0.0
	G-WPI	-0.001	0.050	0.051	95.6	0.005	0.102	0.104	95.0	0.005	0.114	0.117	95.9	-0.001	0.084	0.085	95.8	0.0
	G$^{\mathtt{I}}$-WPI	-0.002	0.045	0.047	96.0	0.002	0.091	0.094	95.6	0.007	0.111	0.115	96.0	-0.000	0.082	0.084	96.4	0.0
PWC-3$^\mathrm{a}$	G-W	-0.003	0.045	0.046	95.6	0.014	0.075	0.078	96.5	0.021	0.092	0.093	95.6	0.007	0.063	0.063	95.0	0.0
	G-WPI	-0.003	0.050	0.051	95.3	0.030	0.108	0.110	94.4	0.040	0.135	0.135	94.2	0.011	0.090	0.092	95.9	0.0
	G$^{\mathtt{I}}$-WPI	-0.004	0.045	0.046	95.7	0.019	0.095	0.098	95.0	0.036	0.126	0.128	94.9	0.010	0.087	0.088	95.5	0.0

$^\mathrm{a}$ Empirical properties are summarised based on replicates leading to convergence for the piecewise constant model. The percentages of replicates failing to converge (out of $nsim=1000$) are indicated in the last column (all replicates converged for the approach based on the correct Weibull marginal model)

$^\mathrm{b}$ G$^\mathtt{I}$ corresponds to $G_i$ with $G_{i21}=\partial \eta _i /\partial \theta ' = 0$; G$^\mathtt{II}$ corresponds to $G_i$ with $G_{i12} = 0$ also; WPI represents the working partial independence assumption with $W_{i22} = \mathrm{diag}\{\eta _i (1-\eta _i)\}$, $W_{i12} = W'_{i21} = 0$

Under more general association structure, results under G$^\mathrm{II}$-WPI estimating equation are not summarized because of high non-convergence percentage for such more general association structure. For the other three conditional estimating equations, their performance was again excellent under the correct Weibull model and again 100% of the replicates lead to convergence for $m=200$ and $m=1000$; see Table 2. Empirical biases were generally small, there was a good agreement between the empirical and average robust standard errors, and the empirical coverage probability was generally within the acceptable range. Under the piecewise constant model, convergence rate was 100% when $m=1000$ and the empirical properties of the estimators for $\beta$ and $\gamma$ were good in such settings. When $m=200$, performance remained good but with small finite sample bias and good empirical coverage probability.

## Impact of Misspecifying the Dependence Structure 

### Limiting Bias Under Misspecified Conditional Estimating Equations

While standard GEE1 only requires correct specification of the marginal mean for consistent estimation of the marginal parameters, the conditional estimating equations require correct specification of the marginal distribution and the dependence structure for consistent estimation, even for the simplified conditional estimating equations G$^\mathrm{I}$-WPI and G$^\mathrm{II}$-WPI. As is often the case, the efficiency gains coming from the use of higher-order moments in the conditional estimating equations such as G–W come at the cost of poorer robustness. We explore the limiting behavior of estimators from misspecified models here based on large sample theory [31]. Specifically, if $U(\psi )$ is an estimating function for $\psi$ based on a misspecified model, then the solution $\widehat{\psi }$ for $U(\psi ) = 0$ asymptotically follows:

5 $$\begin{aligned} \sqrt{m}(\widehat{\psi } - \psi ^{*}) \sim N\left( 0, \bar{\mathcal{A}}^{\,-1}(\psi ^*)\bar{\mathcal{B}}(\psi ^*)[\bar{\mathcal{A}}^{\, -1}(\psi ^*)]'\right) \; \end{aligned}$$

as $m\rightarrow \infty$, where $\bar{\mathcal{A}}(\psi ) = E [-\partial U_i (\psi )/\partial \psi ' \, ; \zeta ]$, $\bar{\mathcal{B}}(\psi ) = E [U_i (\psi ) U'_i(\psi )\, ; \zeta ]$, and $\psi ^*$ is the solution to $E [U(\psi )\, ; \zeta ] = 0$, where $E [\, \cdot \, ; \zeta ]$ denotes an expectation taken with respect to the true distribution indexed by $\zeta$. Note that $E [U(\psi ); \zeta ]$ can be written as

6 $$\begin{aligned} \sum _{i=1}^{m} E \{ U_i (\psi )\, ; \zeta \}= & {} \sum _{i=1}^{m} E\left\{ G'_i\, W^{-1}_i \left( \begin{array}{c} \mu ^{*}_i (\zeta ) -\mu _i \\ \eta _i^{*} (\zeta ) - \eta _i \end{array}\right) + D'_i V_i^{-1}(T_{i0}-\mu _{i0}) \right\} , \end{aligned}$$

where $\mu _i^{*}(\zeta ) = E[ \bar{Y}_i|T_{i0}, X_i, C_i ]$ and $\eta _i^{*}(\zeta ) = E[ \bar{Z}_i|T_{i0}, X_i, C_i ]$ are the conditional expectations of $\bar{Y}_i$ and $\bar{Z}_i$ given $\{T_{i0}, X_i, C_i \}$ under the true model. The expectation on the right-hand side of (6) is taken with respect to the remaining random variables $\{T_{i0}, X_i, C_i\}$. Of course, when the model is correctly specified, then $\psi ^* = \zeta$ but this is not the case more generally; we investigate the limiting bias of estimators under the misspecified model by examining $\psi ^* - \zeta$.

**Fig. 2:**
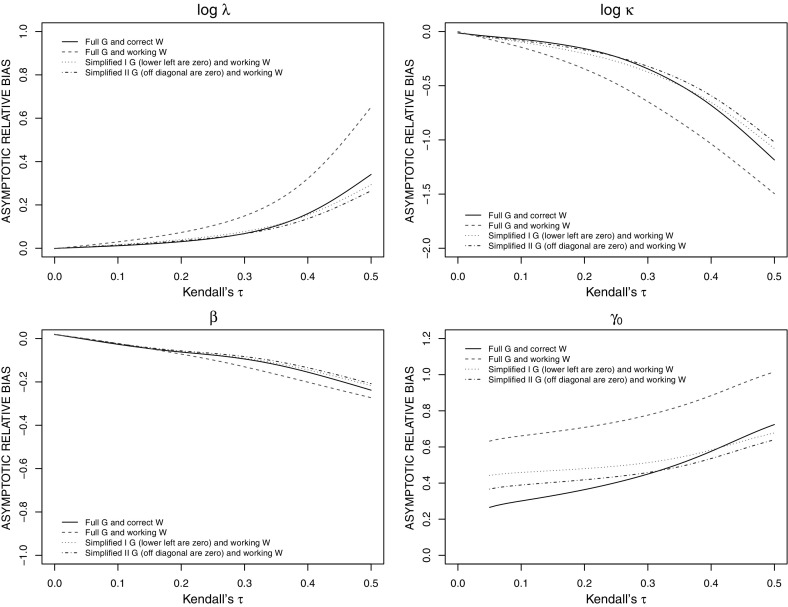
Asymptotic relative biases of estimators under conditional estimating equations when a Gaussian copula with an exchangeable structure is adopted for within-family dependence modeling; the true within-family dependence structure is induced by a Clayton copula; $(\log \lambda , \log \kappa , \beta ) = (-4.11, \log 1.2, \log 1.2)$

Here we consider two-generation families composed of two parents and two children, and the proband is randomly selected from the four family members. The probands are recruited into the registry only if $T_{i0} \le C_{i0}$. We adopt the same parameter settings as in Sect. 3.1, but assume here that the true within-family association structure is induced by the Clayton copula

7 $$\begin{aligned} H(k_0, k_1, \ldots , k_{n_i}\, ; \phi ) = \Big (k_0^{-\phi } + k_1^{-\phi } + \cdots + k_{n_i}^{-\phi } - n_i \Big )^{- 1/\phi }, \end{aligned}$$

where Kendall’s $\tau = \phi / (\phi + 2)$. The adopted estimating functions are misspecified in that the dependence structure is modeled based on a Gaussian copula with an exchangeable association structure. We consider the values of Kendall’s $\tau$ ranging from 0 to 0.5 to reflect independence to strong within-family dependence. We evaluate the limiting relative biases of estimators using Monte Carlo methods to take the expectation in (6) and solving the resulting equation.

From Fig. 2, we see that the conditional estimating equations with the full $G_i$ and WPI matrix $W_i$ is the most sensitive to misspecification. Although one might anticipate that the full $G_i$ and full covariance matrix $W_i$ (G–W) would be less robust than G$^\mathrm{I}$-WPI or G$^\mathrm{II}$-WPI, the asymptotic relative biases of estimators defined through G–W are in general no larger than those under G$^\mathrm{I}$-WPI and G$^\mathrm{II}$-WPI when Kendall’s $\tau$ is less than 0.3; the sensitivity of estimators from G–W to misspecification becomes more apparent, compared to those based on G$^\mathrm{I}$-WPI and G$^\mathrm{II}$-WPI, when Kendall’s $\tau$ is larger (i.e., $>0.3$); G$^\mathtt{I}$-WPI is slightly more sensitive to this form of misspecification than G$^\mathrm{II}$-WPI. Furthermore, the asymptotic relative biases for $\beta$ under the conditional estimating equations are all relatively modest when Kendall’s $\tau$ is small to modest. If one is primarily interested in the estimation of $\beta$, then the proposed conditional estimating equations are reasonably robust to misspecification of the copula function for modest Kendall’s $\tau$, but the asymptotic biases of the dependence parameters are appreciable under misspecification of the dependence structure. This conclusion is analogous to those made regarding misspecification of the random effect distribution with response-dependent sampling [11, 13, 23]. We also conducted supplementary simulation studies demonstrating a good agreement between the finite sample and asymptotic biases in studies with 200 and 1000 families (see Supplementary Material).

### Power Implications of Dependence Structure Misspecification 

We next investigate the effect of dependence structure misspecification on the power of tests regarding covariate effects. Based on our previous findings regarding asymptotic relative efficiency and robustness, here we focus our attention on the preferred estimating functions G–W and G$^\mathrm{I}$-WPI. We consider a test of $H_0 : \beta = \beta _0 = 0$ versus $H_A : \beta \ne 0$, and let $\beta _A$ be the clinically important effect. When both the marginal and association models are correctly specified from (2), we have

8 $$\begin{aligned} \sqrt{m}(\widehat{\beta } - \beta ) \xrightarrow {d} N(0, \sigma ^2 (\psi ) ) , \end{aligned}$$

as $m \rightarrow \infty$, where $\sigma ^2 (\psi )$ is the diagonal element in the robust covariance matrix $\mathcal{A}^{-1}(\psi ) \mathcal{B}(\psi ) \left[ \mathcal{A}^{-1}(\psi )\right] '$ corresponding to $\beta$. Under a two-sided Wald test with significance level 100$\alpha _1$%, the required number of families to ensure 100($1-\alpha _2$)% power to detect $\beta _A$ is the smallest *m* satisfying

9 $$\begin{aligned} m \ge \left\{ \frac{z_{\alpha _1 / 2} ~\sigma (\psi _0) + z_{\alpha _2} ~\sigma (\psi _A)}{\beta _A}\right\} ^2 , \end{aligned}$$

where $\sigma (\psi _0)$ and $\sigma (\psi _A)$ are the square roots of asymptotic variances of $\sqrt{m}(\widehat{\beta }-\beta )$ under the null and alternative hypotheses; $\psi _0 = (\lambda , \kappa , \beta _0, \gamma ')$ and $\psi _A = (\lambda , \kappa , \beta _A, \gamma ')$. $z_u$ is the $100(1-u)\%$ percentile of standard normal distribution.

When the dependence structure is misspecified, the limiting value of estimators under the conditional estimating equations is $\psi ^* (\ne \zeta )$ (Sect. 4.1). Then based on (6), we can calculate the limiting values of $\widehat{\psi }$ under the null and alternative hypotheses when the dependence structure is misspecified, and denote them as $\psi ^{*}_0$ and $\psi ^{*}_A$, respectively. Furthermore, we can show that under the null hypothesis the estimator based on the misspecified conditional estimating equations satisfies

10 $$\begin{aligned} \sqrt{m}(\widehat{\psi } - \psi ^{*}_0) \xrightarrow {d} N(0, \Gamma _0^{*}), \end{aligned}$$

as $m \rightarrow \infty$, and under the alternative hypothesis

11 $$\begin{aligned} \sqrt{m}(\widehat{\psi } - \psi ^{*}_A) \xrightarrow {d} N(0, \Gamma _A^{*}), \end{aligned}$$

where

$$\begin{aligned} \Gamma _0^{*}= & {} \bar{\mathcal{A}}^{\,-1}(\psi ) \bar{\mathcal{B}}(\psi ) [\bar{\mathcal{A}}^{\,-1}(\psi )]'\bigg |_{\psi =\psi _0^{*}} ,\mathrm{and} \Gamma _A^{*}= \bar{\mathcal{A}}^{\,-1}(\psi ) \bar{\mathcal{B}}(\psi ) [\bar{\mathcal{A}}^{\,-1}(\psi )]'\bigg |_{\psi =\psi _A^{*}}. \end{aligned}$$

Hence, the asymptotic properties of $\widehat{\beta }$ can be determined by considering the corresponding component of $\widehat{\psi }$. When the copula model is misspecified, the actual power of such two-sided Wald test of $H_0 : \beta = \beta _0 = 0$ versus $H_A: \beta \ne 0$, at the clinically important effect $\beta _A$ given sample size *m* and significance level $\alpha _1$, is

12 $$\begin{aligned} \mathrm{POWER}= & {} \Phi \left( \frac{- z_{\alpha _1 / 2}\, \sigma ^{*}_0 - \sqrt{m}~\beta ^{*}_A}{\sigma ^{*}_A}\right) + \Phi \left( \frac{-z_{\alpha _1 / 2}\, \sigma ^{*}_0 + \sqrt{m}~\beta ^{*}_A}{\sigma ^{*}_A}\right) , \end{aligned}$$

where $\sigma ^{*}_0$ and $\sigma ^{*}_A$ are the square roots of the diagonal elements of $\Gamma ^{*}_0$ and $\Gamma ^{*}_A\,$, respectively, corresponding to $\beta$.

**Fig. 3:**
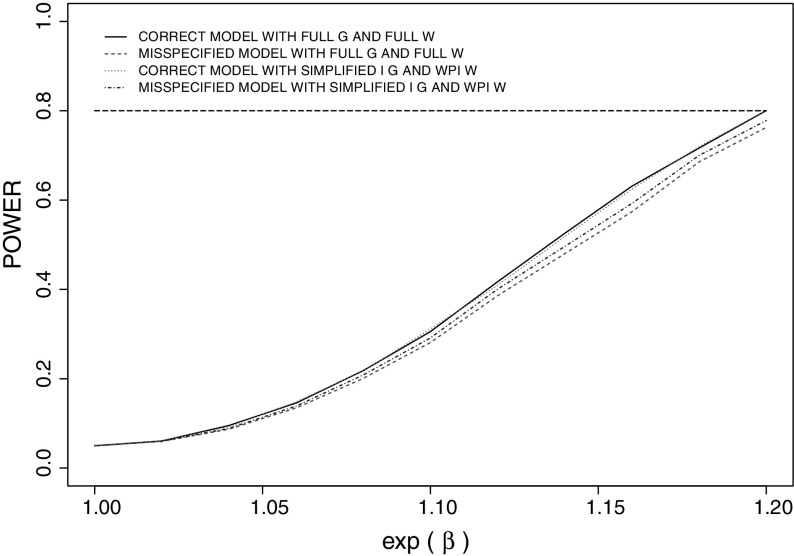
Power curves of a two-sided Wald test for $H_0 : \beta = 0$ under conditional estimating equations G–W and G$^\mathrm{I}$-WPI when the within-family dependence structure is correctly specified or misspecified; true within-family dependence is induced by Gaussian copula with exchangeable structure or Clayton copula, and adopted family dependence structure in the design stage is Gaussian copula with exchangeable association; Kendall’s $\tau = 0.4$, $\beta _A = \log 1.2$

Here we report on an asymptotic study to examine the effect of copula misspecification on the power. Assume that each family consists of two parents and two children, and the proband is randomly selected from the four family members. As before we presume that the families are recruited to the study only if $T_{i0} \le C_{i0}$. The parameter settings are the same as in Sect. 3.1 but we consider two specific scenarios: (i) the true within-family association structure is based on a Gaussian copula with an exchangeable association structure and (ii) the true within-family association structure is based on a Clayton copula (7); in both cases, we set Kendall’s $\tau = 0.4$. At the design stage, we adopt a Gaussian copula with an exchangeable association structure for the within-family dependence, and let Kendall’s $\tau = 0.4$. We therefore only consider the case in which the form of the dependence structure is misspecified. In this setting, we calculate the required sample size to achieve 80% power to reject $H_0$ at $\beta _A = \log 1.2$ by (9), where $\sigma (\psi )$ is obtained from the Gaussian copula. The minimum numbers of families are 420 and 422 based on estimating equation G–W and G$^\mathrm{I}$-WPI, respectively. Under these sample sizes, the actual power of such a design can be computed by (12) for the values of $\beta$ ranging from 0 to $\log 1.2$. The power curves are plotted in Fig. 3 from which we infer that when the association model is correctly specified, tests based on the conditional estimating equations G–W and G$^\mathrm{I}$-WPI have the desired power at the clinically important effect; as expected, the power decreases when the true value of $\beta$ approaches 0. When the copula is misspecified (i.e., the true dependence structure is set by a Clayton copula but a Gaussian copula is used for sample size calculation), tests based on both conditional estimating equations lead to a loss in power, with a greater loss in power under G–W compared to G$^\mathrm{I}$-WPI. This is reasonable since the G–W estimating equations exploit information from higher-order dependencies more than G$^\mathrm{I}$-WPI, which is less robust than the latter. In summary, based on the comprehensive investigation of these conditional estimating equations in terms of efficiency and robustness, estimating equation G$^\mathrm{I}$-WPI is suitable in the absence of information about the association structure, but if information is available about the structure, estimating equation G–W could be adopted to achieve higher efficiency.

## Application to The Psoriatic Arthritis Family Study

Hereditary factors are thought to be important in psoriatic arthritis, as some studies have suggested that close blood relatives of affected individuals are at higher risk of developing the disease compared to the general population. Interest therefore lies in characterizing the effect of genetic markers on the risk of disease; we consider four human leukocyte antigen (HLA) markers reported in the literature as being associated with psoriasis or psoriatic arthritis including HLA-B8, HLA-B27, HLA-C6, and HLA-C12. Characterizing the nature of the within-family association structure can also provide useful insight into the genetic basis of the disease. Particular interest lies in assessing the “parent-of-origin” effect; preliminary evidence suggests that there may be a stronger risk of paternal transmission, over maternal transmission, of risk of disease; we refer readers to Pollock et al. [24] for associated results based on binary analyses.

Here we consider an application to the motivating family study on the genetic basis of psoriatic arthritis conducted in the Centre for Prognosis Studies in the Rheumatic Diseases at the University of Toronto. One hundred and sixty-nine families composed of 2 to 7 members, including the proband, were recruited for study. The date of disease onset is available for probands from the clinic registry but only the disease status of other individuals is available when they are examined, yielding current status data. A Weibull model is adopted for the marginal distribution of the PsA onset time with survivor function $\mathcal{F}(t|X_{ij}; \theta ) = \exp (- (\lambda t)^{\kappa }\exp (X'_{ij}\beta ))$ where $\theta = (\lambda , \kappa , \beta ')'$, $j=1, \ldots , n_i$, and $i=1, \ldots , 169$. A flexible model for the within-family dependence is formulated based on a Gaussian copula with different pairwise dependencies between parents ($\tau _\mathrm{pp}$), between siblings ($\tau _\mathrm{ss}$), between a father and his child ($\tau _\mathrm{fc}$), and between a mother and her child ($\tau _\mathrm{mc}$). This can be formulated in terms of a second-order regression model given by

13 $$\begin{aligned} \log \big ((1+\tau _{ijk})/(1-\tau _{ijk})\big ) = \gamma _0 + \gamma _1 v_{ijk1} + \gamma _{2} v_{ijk2} + \gamma _3 v_{ijk3} , \end{aligned}$$

where $v_{ijk1} = I((j, k)~\mathrm{pair~are~siblings})$, $v_{ijk2}=I((j,k)~\mathrm{pair~is~father{-}child})$, and $v_{ijk3}=I((j,k)~\mathrm{pair~is~mother{-}child})$. The hypotheses $H_0 : \gamma _2 - \gamma _3 = 0$ and $H_A : \gamma _2 - \gamma _3 \ne 0$ are the basis of a test regarding the parent-of-origin question. There are only 8 pairs of parents, which leads to insufficient data to estimate the intercept in (13) and so we constrain that parameter to be zero with the implicit assumption that there are no environmental determinants of PsA.

Table 3 summarizes the results with the top half obtained from the full derivative and covariance matrices (G–W) and the bottom half reporting the results from G$^\mathrm{I}$-WPI. A model with no HLA covariate is given in the first column followed by four univariate models, with the last column containing results from a multivariate model including all four markers. The estimates for the association parameters are given in terms of $\gamma$ and the three Kendall’s $\tau$ parameters.

Based on the model with no HLA covariates, we find $\hat{\tau }_\mathrm{ss} = 0.337$ (95% CI: 0.113, 0.528; *p* value = 0.002), indicating highly significant association between siblings in the disease onset time. The father–child association is lower at $\hat{\tau }_\mathrm{fc} = 0.225$ (95% CI: −0.030, 0.452) and not quite statistically significant (*p* value = 0.072). For the mother–child association, we find $\hat{\tau }_\mathrm{mc} = 0.130$ (95% CI: −0.153, 0.393) which is weaker still and insignificant (*p* value = 0.364). A test of the parent-of-origin hypothesis based on $H_0 : \gamma _2 - \gamma _3 = 0$ yields a Wald statistic of 1.435 (*p* value = 0.151). As this is not statistically significant at the 5% significance level, there is insufficient evidence to claim a statistically significant “parent- of-origin” effect. The results are broadly comparable for the HLA regression analyses based on the other conditional estimating equation (G$^\mathrm{I}$-WPI). For the association parameters, the estimates are somewhat lower with $\hat{\tau }_\mathrm{ss} = 0.220$ (95% CI: −0.003, 0.423; *p* value = 0.046), $\hat{\tau }_\mathrm{fs} = 0.104$ (95% CI: −0.128, 0.324; *p* value = 0.378), and $\hat{\tau }_\mathrm{ms}= -0.018$ (95% CI: −0.256, 0.222; *p* value = 0.886). The Wald statistic of 1.682 (*p* value = 0.092) does not suggest a “parent- of-origin” effect.

The large sample theory we develop can be used to plan a future family study and it is possible to calculate how many families would be required to ensure adequate power to test the parent-of-origin hypothesis in a future study. In a new study, we may consider recruitment of families of members of the registry and presume that the distribution of family members, ages at assessment, and other factors are similar in the new study. We use the sample size formula similar to (9) but for $\gamma _2 - \gamma _3$ and determine that 627 families would be required to ensure 80% power to detect a significant difference between the father–child and mother–child associations using estimating function G–W when the true effects correspond to those seen in the first column of Table 3. The current study therefore appears to be grossly under-powered to formally test the parent-of-origin hypothesis.

None of the HLA markers were shown to have a significant effect on the time to the onset of PsA. Based on the G–W estimating equations, there is a trend toward a reduction in risk with HLA-B8 and a trend toward an increased risk with the presence of each of the other HLA markers.

**Table 3:** Estimates of analyses of HLA markers and time to disease onset based on conditional estimating equations using response-biased psoriatic arthritis family data; associated standard errors are in the parentheses

	Univariate models					Multivariate model
	No HLA	HLA-B8	HLA-B27	HLA-C6	HLA-C12	
Full G and Full W (G$-$W)						
$\log \lambda$	$-$5.461 (0.531)	$-$5.353 (0.455)	$-$5.573 (0.609)	$-$5.650 (0.590)	$-$5.342 (0.464)	$-$5.544 (0.543)
$\log \kappa$	1.347 (0.086)	1.347 (0.086)	1.348 (0.086)	1.349 (0.086)	1.346 (0.087)	1.349 (0.086)
$\beta _{B8}$	$-$	$-$0.483 (0.709)	$-$	$-$	$-$	$-$0.195 (0.751)
$\beta _{B27}$	$-$	$-$	1.161 (0.755)	$-$	$-$	1.308 (0.771)
$\beta _{C6}$	$-$	$-$	$-$	0.397 (0.593)	$-$	0.458 (0.625)
$\beta _{C12}$	$-$	$-$	$-$	$-$	0.788 (0.769)	0.982 (0.830)
$\gamma _1$	0.700 (0.242)	0.660 (0.224)	0.741 (0.261)	0.777 (0.242)	0.620 (0.234)	0.695 (0.244)
$\gamma _2$	0.458 (0.264)	0.414 (0.242)	0.470 (0.281)	0.536 (0.259)	0.378 (0.264)	0.423 (0.269)
$\gamma _3$	0.261 (0.291)	0.220 (0.268)	0.300 (0.310)	0.343 (0.285)	0.173 (0.289)	0.252 (0.287)
$\tau _{ss}$	0.337 (0.107)	0.318 (0.101)	0.354 (0.114)	0.370 (0.104)	0.301 (0.106)	0.334 (0.108)
$\tau _{fc}$	0.225 (0.125)	0.204 (0.116)	0.231 (0.133)	0.262 (0.121)	0.187 (0.127)	0.209 (0.129)
$\tau _{mc}$	0.130 (0.143)	0.110 (0.132)	0.149 (0.151)	0.170 (0.138)	0.086 (0.143)	0.125 (0.141)
G$^{\text{ I }}$ and WPI W (G$^{\text{ I }}$ $-$WPI)						
$\log \lambda$	$-$5.037 (0.326)	$-$5.007 (0.313)	$-$5.105 (0.358)	$-$5.034 (0.325)	$-$5.052 (0.317)	$-$5.105 (0.331)
$\log \kappa$	1.337 (0.087)	1.338 (0.087)	1.339 (0.087)	1.337 (0.087)	1.338 (0.088)	1.340 (0.087)
$\beta _{B8}$	$-$	$-$0.490 (0.644)	$-$	$-$	$-$	$-$0.325 (0.669)
$\beta _{B27}$	$-$	$-$	0.849 (0.587)	$-$	$-$	0.969 (0.580)
$\beta _{C6}$	$-$	$-$	$-$	$-$0.037 (0.492)	$-$	0.060 (0.537)
$\beta _{C12}$	$-$	$-$	$-$	$-$	0.767 (0.644)	0.911 (0.667)
$\gamma _1$	0.448 (0.232)	0.447 (0.234)	0.481 (0.237)	0.447 (0.238)	0.437 (0.227)	0.465 (0.238)
$\gamma _2$	0.208 (0.237)	0.199 (0.234)	0.208 (0.238)	0.206 (0.241)	0.195 (0.241)	0.180 (0.244)
$\gamma _3$	$-$0.036 (0.249)	$-$0.039 (0.246)	$-$0.005 (0.250)	$-$0.037 (0.256)	$-$0.059 (0.251)	$-$0.036 (0.254)
$\tau _{ss}$	0.220 (0.110)	0.220 (0.111)	0.236 (0.112)	0.220 (0.113)	0.215 (0.108)	0.228 (0.113)
$\tau _{fc}$	0.104 (0.117)	0.099 (0.116)	0.104 (0.118)	0.103 (0.119)	0.097 (0.119)	0.090 (0.121)
$\tau _{mc}$	$-$0.018 (0.124)	$-$0.019 (0.123)	$-$0.002 (0.125)	$-$0.019 (0.128)	$-$0.030 (0.125)	$-$0.018 (0.127)

## Discussion

Estimating functions have been developed to model the nature and extent of within-family dependence in disease onset times from family studies under response-dependent sampling. A novel aspect of this work is the formulation of the dependence measures on the basis of the disease onset time and the recognition that the available data on family members are handled more naturally as current status data rather than binary data. This approach utilizes all available data from probands and their relatives in assessing the association between age of onset and covariates and in evaluating the association structure of age of onset among family members. The biased sampling scheme typically employed in family studies is addressed by the use of conditional estimating equations where the conditioning event reflects the selection criteria. Several specific estimating functions within the class proposed are assessed in terms of efficiency and robustness; these results complement the standard results of second-order estimating functions since all moments in the proposed equations are conditional. We also outline how sample size requirements for family studies can be assessed based on this framework to ensure that power objectives are met. Code for solving the conditional second-order estimating equations (1) and for obtaining the variance estimates of Sect. 2.2 are available at Github https://github.com/Yujie-Zhong/CGEE2.

We have focused on the use of estimating functions for the analysis of family data in part because the likelihood can be challenging to compute when the size of the family is large. Nevertheless, some assessment of the loss of efficiency in comparison to this optimal approach would be worthwhile. The validity of the proposed conditional second-order estimating equations hinges on correct specification of the dependence structure, a requirement that is analogous to the need for correct specification of the mixing distribution in random effects models for data obtained based on a response-dependent sampling scheme [23]. Assessing model adequacy is best done by testing for the need for model expansion; this could be carried out by testing the need for more cut-points in the baseline hazard function to accommodate a more flexible hazard function, or the need to test for a more general dependence structure. In the present setting, the dependence structure is most easily formulated by selecting a working copula model for the joint distribution of the onset times in the population. If this dependence structure is misspecified, inconsistent estimates are obtained, and we examine their consequences in Sect. 4 to make recommendations on the use of a particular derivative and working covariance matrix. The properties of estimators under model misspecification can be explored using large sample theory [31], but these will be influenced by response-dependent sampling schemes and so a more general study of the effect of misspecification in this framework represents an important area for further research.

We have restricted our attention to parametric models for the onset time distribution. Natural extensions would be to introduce non-parametric or semi-parametric methods for estimating the marginal distributions. In the latter case, one can look at multiplicative Cox models, accelerated failure time models, and Aalen’s additive model, among many other methods. Joint estimation based on the most general conditional estimating equation can be challenging in this setting, but two-stage estimation procedures may be feasible; this is an area of current research. The preliminary work based on the piecewise constant baseline hazard model, however, suggests that studies may need to recruit a lot of families if the incidence rate is low to estimate the marginal onset time distribution. If the disease onset times are available for all or even some of the non-probands found to have the disease, these data could help in estimation; the estimating equations we present can be modified in this case to incorporate such data. Auxiliary samples can also be useful to enhance inferences.

While there is an increasing amount of attention given to the use of disease onset time as a basis for modeling within-family dependence, there remain challenging issues that warrant further attention. The primary challenge is in quantifying dependence in the presence of the competing risk of death [12, 26]. The classical illness–death process is a natural framework for modeling the occurrence of disease in individuals who are at risk, and generalization of this set-up to model within-family dependence is an area warranting attention. This issue is not unique to analyses based on disease onset times; when current status data are treated as binary data, the requirement that individuals are alive at the time of contact is ignored.

## Electronic supplementary material

Below is the link to the electronic supplementary material.

Supplementary material 1 (pdf 53 KB)
