# Supplementary material for: Second-Order Estimating Equations for Clustered Current Status Data from Family Studies Using Response-Dependent Sampling
Source: Stat Biosci. 2017 Jul 24;10(1):160–83. doi: 10.1007/s12561-017-9201-4 (PMC6097126; doi:10.1007/s12561-017-9201-4)
Supplement: Supplementary file 1 — Supplementary material 1 (pdf 53 KB) [file 12561_2017_9201_MOESM1_ESM.pdf]

Noname manuscript No.
(will be inserted by the editor)

Supplementary Material for

Second-order estimating equations for clustered current status data from family studies using response-dependent sampling

Yujie Zhong · Richard J. Cook

Received: date / Accepted: date

1 Finite sample study of the conditional estimating equations under misspecification

We investigate effect of the misspecified model when using conditional estimating equations in Section 4.1 and point out how to examine the limiting behaviour of estimators under misspecification.

To illustrate it, we consider a two-generation families comprised of two parents and two children. A Gaussian copula with an exchangeable structure is adopted for within-family dependence modeling while the true within-family dependence structure is induced by a Clayton copula. Limiting bias of estimators using the proporsed conditional estimating equations are calculated as a function of the strength of the within-family association, and their asymptotic relative bias are shown in Figure 2. We also conduct supplementary simulation studies to examine the finite sample performance and to confirm the agreement. The simulation results are therefore reported in Table S.1.

Y. Zhong
MRC Biostatistics Unit, School of Clinical Medicine, University of Cambridge, Cambridge Institute of Public Health, Forvie Site, Robinson Way, Cambridge CB2 0SR, UK
E-mail: yujie.zhong@mrc-bsu.cam.ac.uk

R.J. Cook
Department of Statistics and Actuarial Science, University of Waterloo, Waterloo, ON, N2L 3G1, Canada

Table S.1 Empirical properties of estimates under conditional estimating equations for family data from response-dependent sampling using Gaussian copula with exchangeable association structure to model the within-family association, where true within-family dependence structure is induced by Clayton copula; $n_i = 3$, $nsim = 1000$.

		$\log\lambda$					$\log\kappa$					β					γ_0				
τ	EE†	BIAS[1]	BIAS[2]	ESE	ASE	ECP	BIAS[1]	BIAS[2]	ESE	ASE	ECP	BIAS[1]	BIAS[2]	ESE	ASE	ECP	BIAS[1]	BIAS[2]	ESE	ASE	ECP
											$m = 1000$										
0.0	G-W	-0.001	0.000	0.037	0.037	94.3	0.000	0.005	0.032	0.031	94.6	-0.002	0.001	0.049	0.052	96.0	0.000	0.000	0.029	0.029	95.2
	G-WPI	-0.000	0.000	0.046	0.045	94.2	0.000	0.004	0.033	0.032	94.5	-0.002	0.001	0.054	0.056	95.5	-0.001	0.001	0.043	0.043	94.4
	G^{I}-WPI	-0.000	0.000	0.039	0.038	94.7	0.000	0.005	0.032	0.031	94.6	-0.002	0.001	0.049	0.052	96.0	-0.000	0.001	0.035	0.035	94.8
	G^{II}-WPI	-0.001	0.000	0.038	0.038	94.9	0.000	0.005	0.032	0.031	94.5	-0.002	0.001	0.049	0.052	96.1	0.000	0.001	0.034	0.033	94.9
0.2	G-W	-0.132	-0.132	0.066	0.064	46.5	-0.033	-0.028	0.034	0.033	81.9	-0.008	-0.014	0.049	0.048	93.7	0.149	0.150	0.056	0.053	18.4
	G-WPI	-0.318	-0.310	0.111	0.107	9.0	-0.067	-0.062	0.039	0.037	57.0	-0.011	-0.014	0.054	0.052	93.5	0.291	0.290	0.074	0.072	0.5
	G^{I}-WPI	-0.169	-0.170	0.070	0.073	32.4	-0.041	-0.037	0.034	0.034	76.2	-0.008	-0.013	0.049	0.048	94.0	0.195	0.197	0.056	0.060	4.9
	G^{II}-WPI	-0.146	-0.147	0.069	0.071	45.4	-0.035	-0.031	0.034	0.034	81.0	-0.007	-0.013	0.049	0.048	94.0	0.170	0.172	0.058	0.061	16.3
0.4	G-W	-0.677	-0.617	0.258	0.219	0.3	-0.134	-0.113	0.045	0.042	10.8	-0.029	-0.027	0.037	0.037	88.1	0.502	0.474	0.122	0.103	0.0
	G-WPI	-1.406	-1.225	0.570	0.507	0.0	-0.199	-0.178	0.048	0.046	0.9	-0.037	-0.035	0.038	0.038	81.6	0.772	0.728	0.168	0.152	0.0
	G^{I}-WPI	-0.652	-0.611	0.258	0.262	2.9	-0.127	-0.109	0.045	0.048	20.0	-0.026	-0.025	0.037	0.037	89.2	0.504	0.486	0.121	0.126	0.0
	G^{II}-WPI	-0.573	-0.543	0.235	0.256	16.0	-0.115	-0.098	0.045	0.049	32.8	-0.024	-0.024	0.038	0.038	89.9	0.461	0.447	0.121	0.132	0.0
											$m = 200$										
	G-W	-0.000	0.000	0.080	0.082	94.6	0.003	0.005	0.069	0.068	95.5	0.002	0.001	0.113	0.115	95.5	-0.002	0.000	0.067	0.065	94.1
	G-WPI	-0.006	0.000	0.105	0.103	95.7	0.003	0.004	0.073	0.072	94.8	0.002	0.001	0.124	0.125	95.7	0.001	0.001	0.102	0.096	93.5
	G^{I}-WPI	-0.001	0.000	0.084	0.085	94.6	0.004	0.005	0.069	0.069	95.4	0.001	0.001	0.113	0.115	95.6	-0.001	0.001	0.081	0.077	93.1
	G^{II}-WPI	-0.001	0.000	0.083	0.085	94.2	0.003	0.005	0.069	0.069	95.5	0.001	0.001	0.113	0.115	95.7	-0.001	0.001	0.079	0.074	93.1
0.2	G-W	-0.148	-0.132	0.148	0.149	93.5	-0.032	-0.028	0.073	0.073	93.1	-0.006	-0.014	0.110	0.108	95.5	0.153	0.150	0.125	0.119	80.2
	G-WPI	-0.363	-0.310	0.276	0.263	92.4	-0.067	-0.062	0.084	0.082	88.2	-0.009	-0.014	0.118	0.116	94.2	0.303	0.290	0.175	0.166	58.1
	G^{I}-WPI	-0.189	-0.170	0.162	0.172	95.3	-0.040	-0.037	0.075	0.076	93.0	-0.006	-0.013	0.110	0.107	95.1	0.200	0.197	0.132	0.136	75.3
	G^{II}-WPI	-0.166	-0.147	0.158	0.169	97.2	-0.035	-0.031	0.074	0.075	94.1	-0.006	-0.013	0.110	0.108	95.1	0.176	0.172	0.134	0.138	83.6
0.4	G-W	-0.791	-0.617	0.762	0.626	99.8	-0.123	-0.113	0.096	0.092	75.9	-0.025	-0.027	0.084	0.084	93.0	0.508	0.474	0.280	0.239	40.8
	G-WPI	-1.514	-1.225	1.205	1.354	100.0	-0.177	-0.178	0.099	0.100	59.8	-0.030	-0.035	0.089	0.086	91.4	0.750	0.728	0.319	0.341	26.4
	G^{I}-WPI	-0.765	-0.611	0.777	0.806	100.0	-0.115	-0.109	0.094	0.104	86.8	-0.024	-0.025	0.085	0.084	93.0	0.508	0.486	0.272	0.299	82.2
	G^{II}-WPI	-0.722	-0.543	0.830	0.870	100.0	-0.105	-0.098	0.097	0.107	90.8	-0.022	-0.024	0.085	0.085	93.6	0.475	0.447	0.291	0.320	98.2

BIAS[1] is the averaged bias of the estimates and BIAS[2] is the limiting bias of the estimates;
† G^{I} corresponds to G_i with $G_{i21} = \partial\eta_i/\partial\theta' = 0$; G^{II} corresponds to G_i with $G_{i12} = 0$ also;
WPI is working partial independence assumption with $W_{i22} = \text{diag}\{\eta_i(1-\eta_i)\}$, $W_{i12} = W'_{i21} = 0$.
